# VISTA expression by microglia decreases during inflammation and is differentially regulated in CNS diseases

**DOI:** 10.1002/glia.23517

**Published:** 2018-10-11

**Authors:** Malte Borggrewe, Corien Grit, Wilfred F. A. Den Dunnen, Saskia M. Burm, Jeffrey J. Bajramovic, Randolph J. Noelle, Bart J. L. Eggen, Jon D. Laman

**Affiliations:** ^1^ Department of Neuroscience, Section Medical Physiology University of Groningen, University Medical Center Groningen Groningen The Netherlands; ^2^ Department of Pathology, University of Groningen University Medical Center Groningen Groningen The Netherlands; ^3^ Alternatives Unit, Biomedical Primate Research Centre Rijswijk The Netherlands; ^4^ Department of Microbiology and Immunology Geisel School of Medicine at Dartmouth, Norris Cotton Cancer Center Lebanon New Hampshire

**Keywords:** autoimmunity, cancer, DD1alpha, immune checkpoints, immunotherapy, neurodegeneration, PD‐1H

## Abstract

V‐type immunoglobulin domain‐containing suppressor of T‐cell activation (VISTA) is a negative checkpoint regulator (NCR) involved in inhibition of T cell‐mediated immunity. Expression changes of other NCRs (PD‐1, PD‐L1/L2, CTLA‐4) during inflammation of the central nervous system (CNS) were previously demonstrated, but VISTA expression in the CNS has not yet been explored. Here, we report that in the human and mouse CNS, VISTA is most abundantly expressed by microglia, and to lower levels by endothelial cells. Upon TLR stimulation, VISTA expression was reduced in primary neonatal mouse and adult rhesus macaque microglia in vitro. In mice, microglial *VISTA* expression was reduced after lipopolysaccharide (LPS) injection, during experimental autoimmune encephalomyelitis (EAE), and in the accelerated aging *Ercc1* ^*Δ/−*^ mouse model. After LPS injection, decreased *VISTA* expression in mouse microglia was accompanied by decreased acetylation of lysine residue 27 in histone 3 in both its promoter and enhancer region. ATAC‐sequencing indicated a potential regulation of *VISTA* expression by Pu.1 and Mafb, two transcription factors crucial for microglia function. Finally, our data suggested that VISTA expression was decreased in microglia in multiple sclerosis lesion tissue, whereas it was increased in Alzheimer's disease patients. This study is the first to demonstrate that in the CNS, VISTA is expressed by microglia, and that VISTA is differentially expressed in CNS pathologies.

## 1 INTRODUCTION

Immune checkpoint regulators are a group of molecules expressed on T cells or antigen‐presenting cells (APCs), which can provide co‐stimulatory and co‐inhibitory signals during T cell activation. This balance between positive and negative signals is essential for mounting antigen specific immune responses, while limiting the risk for autoimmunity. Inhibition of negative checkpoint regulator (NCR) activity has recently entered the clinic as a treatment for cancer, whereas activation of NCRs has potential for the treatment of autoimmunity. Therapeutic inhibition of NCR activity (immunotherapy) in cancer has been associated with the development of central nervous system (CNS) diseases such as encephalitis, myelitis, and exacerbation of multiple sclerosis (MS) (Cuzzubbo et al., 2017; Yshii, Hohlfeld, & Liblau, 2017). Accordingly, several studies have reported that inhibition of NCRs (CTLA4, PD‐1, and PD‐L1) in mouse experimental autoimmune encephalomyelitis (EAE), a model of MS, leads to exacerbation of symptoms (Joller, Peters, Anderson, & Kuchroo, 2012). Furthermore, blockade of PD‐1 in a mouse model of Alzheimer's disease (AD) improves cognitive performance (Baruch et al., 2016), suggesting a broad role of NCRs in CNS pathologies. However, the effectiveness of PD‐1 blockade in AD is still not resolved (Latta‐Mahieu et al., 2018).

V‐domain Ig‐containing suppressor of T cell activation (VISTA) is a recently discovered NCR, which shares 24% sequence similarity with PD‐L1 (Wang et al., 2011). VISTA (aliases: PD‐1H (Flies, Wang, Xu, & Chen, 2011); DD1α (Yoon et al., 2015); Dies1 (Aloia, Parisi, Fusco, Pastore, & Russo, 2010); Gi24 (Sakr et al., 2010)) is expressed on myeloid and T cells, and can act as both receptor and ligand (Lines, Sempere, Broughton, Wang, & Noelle, 2014). VISTA expressed on APC can function as a ligand, suppressing T‐cell activation upon binding to a yet unidentified counter receptor (Wang et al., 2011). In addition, ligation of VISTA expressed on T cells also leads to inhibition of T cell activation (Flies et al., 2014). Receptor functions on myeloid cells include regulation of the cytokine response (Bharaj et al., 2014) and uptake of apoptotic cells (Yoon et al., 2015). Deficiency of VISTA in mice increases susceptibility to developing autoimmunity such as EAE (Wang et al., 2014) and lupus nephritis (Ceeraz et al., 2016).

In the CNS, expression of several NCRs by microglia has been reported, which is induced under inflammatory conditions (Yshii et al., 2017). Microglia are the principal innate immune cell type of the CNS, which acquire diverse functional phenotypes in response to environmental cues (Salter & Beggs, 2014). During CNS pathologies, microglia lose their homeostatic signature and can shift to an immune‐activated state, as evident from transcriptomic studies (Perry & Holmes, 2014; Zrzavy et al., 2017). During immune activation, microglia upregulate inflammatory genes such as genes involved in cytokine production and antigen presentation. It is now appreciated that in conditions such as AD and aging, microglia can acquire a phagocytic phenotype (Galatro et al., 2017a; Krasemann et al., 2017; Varol et al., 2017), thereby facilitating clearance of debris and toxins. In accordance with a more protective phenotype during disease, microglia obtain immunoregulatory characteristics during inflammation. Induction of NCRs (e.g., PD‐L1) in immune‐activated microglia causes inhibition of T cell activation, and suppression of cytokine production (Duncan & Miller, 2011; Magnus, 2005; Schachtele, Hu, Sheng, Mutnal, & Lokensgard, 2014). Hence, microglial activation is highly heterogeneous and plastic, and can therefore be detrimental or beneficial during disease.

Although the expression and regulation of several NCRs in the CNS and by microglia has been studied, expression patterns of VISTA are unknown as noted recently by Yshii et al. (2017). Studies suggest involvement of VISTA in important functions of monocytes and macrophages, such as cytokine responses and phagocytosis, which exhibit functional similarities to microglia. As microglia express many NCRs and are able to acquire immunoregulatory functions during inflammation, analysis of VISTA expression in these cells could help to understand their role in CNS disease. Furthermore, NCR modulation as a treatment for cancer and autoimmunity impacts CNS biology, which is demonstrated by adverse neurological effects during immunotherapy including encephalitis, myelitis, hypophysitis, and transition from radiologically isolated syndrome to MS (Cuzzubbo et al., 2017; Yshii et al., 2017). Hence, investigating expression and expression changes of VISTA in the CNS might facilitate understanding the impact of NCR modulation on the CNS.

Here, we assessed VISTA expression in mouse and rhesus macaque microglia after immune‐activation in vitro and in vivo, and verified our findings using human postmortem tissue. Our results indicate that VISTA is differentially expressed in microglia during inflammation and neurodegeneration. Furthermore, we determined epigenetic changes in the *VISTA* gene during microglial activation in mice. These findings provide first evidence of a function of VISTA in microglia and during CNS pathology.

## 2 MATERIALS AND METHODS

### 2.1 Animals

All animal experiments were approved by the Netherlands Central Committee for Animal Experiments and the University of Groningen. Mice were housed SPF in groups in macrolon cages with ad libitum access to water and food, and a 12 hr light–dark cycle. Eight‐week‐old male C57BL/6 mice (bred in‐house) were injected with 1 mg/kg LPS (*E. coli* 0111:B4, Sigma‐Aldrich, L4391) intraperitoneally and sacrificed 24 hr later. To generate *Ercc1* ^*Δ/−*^ mice, *Ercc1* ^*Δ/+*^ Fvb mice were bred with *Ercc1* ^*+/−*^ C57BL/6 mice. Genotype was confirmed using PCR and *Ercc1* ^*Δ/−*^ mice were matched with wildtype littermates of the same sex. After 3–4 months of age, *Ercc1* ^*Δ/−*^ mice started to develop tremors and aberrant behavior, and were sacrificed. For induction of EAE, 10‐week‐old female C57BL/6 mice (Harlan, The Netherlands) were immunized with MOG_35–55_ in complete Freund's adjuvant (CFA) (Hooke, EK‐2110) and injected with pertussis toxin on the day of immunization and 24 hr later. Mice were monitored daily for development of EAE and sacrificed at score 1 (limp tail), score 4 (complete hind leg paralysis), and remission (partial regain of movement in hind legs).

### 2.2 Acute microglia isolation

Microglia were isolated as described previously (Galatro, Vainchtein, Brouwer, Boddeke, & Eggen, 2017b). Briefly, mice were perfused using PBS (Lonza, BE17‐512F) and brain and spinal cord were isolated in HBSS (Gibco, 14170‐088) containing 15 mM HEPES (Lonza, BE17‐737E) and 0.6% glucose (Sigma‐Aldrich, G8769) (=Medium A). Tissue was mechanically disrupted to obtain a single cell suspension and myelin was removed using 24.4% percoll (GE Healthcare, 17‐0891‐01) density gradient centrifugation at 950g. Cells were incubated 15 min in anti‐Cd16/32 blocking buffer (clone 93, eBioscience, 14‐0161‐85) and stained with anti‐Cd11b‐PE‐Cy7 (clone M1/70, eBioscience, 25‐0112‐81), anti‐Cd45‐FITC (clone 30‐F11, eBioscience, 11‐0451‐85), and anti‐Ly6c‐APC (clone HK1.4, Biolegend, 128016) antibodies 30 min on 4°C. Cells were sorted on a MoFlo XDP Cell Sorter (Beckman Coulter) in RNAlater (Qiagen, 76104) in siliconized tubes. Sorted cells were centrifuged at 5,000g (RNAlater) and lysed in RLT+ lysis buffer (Qiagen, 74034).

### 2.3 Immunohistochemistry

Immunohistochemical staining was performed on formalin‐fixed paraffin‐embedded (FFPE) or paraformaldehyde (PFA)‐fixed frozen tissue as indicated. Briefly, FFPE tissue was deparaffinized in xylene (J.T. Baker, 9490) and rehydrated. For human tissue, sodium citrate (pH 6.0) heat‐induced antigen retrieval was performed in a microwave using a pressure cooker, whereas Tris–EDTA (pH 9.0) was used for mouse tissue. Endogenous peroxidase activity was blocked in 0.3% hydrogen peroxide for 30 min and mouse tissue was additionally blocked in 10% normal serum. Primary antibodies were applied at 4°C overnight (Supporting Information, Table S1). For human tissue, primary antibodies were diluted in Normal Antibody Green Bright Diluent (ImmunoLogic, BD09‐500). Fluorophore‐conjugated (Molecular Probes) or biotinylated (Vector) secondary antibodies were applied for 1 hr at room temperature (RT). For fluorescence staining, tissue was incubated 10 min in Hoechst and human tissue was treated with 0.3% sudan black to quench autofluorescence. Tyramide Superboost streptavidin kit (Invitrogen, B40933) was used for VISTA (clone MH5a) according to manufacturer's instructions. For enzymatic immunostaining, tissue was incubated 30 min in Vectastain Elite ABC‐HRP (Vector, PK‐6100) and immunoreactivity was revealed using 3,3′‐diaminobenzidine.

### 2.4 Primary mouse neonatal microglia culture

Primary neonatal mouse microglia cultures were prepared as described previously (Schaafsma et al., 2015) with minor deviations. Briefly, cerebrum from postnatal day 0–2 C57BL/6 mice was minced and incubated in trypsin‐containing medium. After trituration and centrifugation of tissue, cells were plated in flasks and medium (DMEM (Gibco, 11500416), supplemented with 1 mM sodium pyruvate (Lonza, BE13‐115E), 1× GlutaMAX (Gibco, 35050038), 1% Pen/strep, and 10% FCS (Gibco, 10500064)) was replaced 24 hr later. Medium was replaced on Day 4, and on Day 7, medium supplemented with 33% L929 cell‐conditioned medium (LCCM) was added. LCCM contains M‐CSF to stimulated microglia proliferation. Three days after LCCM addition, microglia were harvested through shake off and seeded at 30,000 cells/cm^2^ in 12‐well plates. Twenty‐four hours after seeding, primary neonatal microglia were simulated with 100 ng/ml Pam3CSK4 (Invivogen, #tlrl‐pms), 100 ng/ml LPS (*E. coli* 0111:B4, Sigma‐Aldrich, L4391), 50 μg/ml PolyI:C (Invivogen, #tlrl‐pic), or 10 μg/ml β‐glucan (Sigma‐Aldrich, G5011).

### 2.5 Primary adult rhesus macaque microglia culture

Primary adult rhesus macaque (*Macaca mulatta*) microglia cultures were isolated from post mortem brain tissue and cultured as described previously (Burm et al., 2015). Briefly, male or female rhesus macaque monkeys (4–9 years old) without neurological symptoms were housed in outbred breeding colonies. No monkey was sacrificed exclusively for the generation of the primary cultures. Cubes of 3 g of subcortical white matter brain tissue were mechanically and enzymatically dissociated and centrifuged. A percoll gradient was used to remove myelin; other CNS cells and erythrocytes were removed by exposure to hypotonic solution. Isolated microglia were cultured in DMEM (high glucose)/HAM F10 Nutrient Mixture (supplemented with 10% v/v heat‐inactivated FCS, 0.5 mm glutamax, 50 U/ml penicillin, and 50 μg/ml streptomycin) (Thermo Fisher). After 24 hr, nonadherent cells and cellular debris were removed and fresh medium containing 20 ng/ml macrophage colony‐stimulating factor (M‐CSF) (Peprotech) was added. After 7 days in culture, microglia were treated for 16 hr with 500 ng/ml Pam3CSK4 (Invivogen), 20 μg/ml Poly I:C (Invivogen), 100 ng/ml LPS (*E. coli* 026:B6, Sigma‐Aldrich, L8274), or 1 μg/ml CL075 (Invivogen, #tlrl‐c75).

### 2.6 Gene expression analysis

For quantitative PCR analysis, total RNA was isolated using TRIzol (Invitrogen, 15596018) (primary microglia) or the RNeasy Micro Plus Kit (Qiagen, 74034) (acutely isolated microglia) according to the manufacturers protocol. Total RNA was reverse‐transcribed using the RevertAid First Strand cDNA Synthesis kit (Thermo Fisher, K1622). Quantitative PCR was performed using exon–exon spanning primers pairs (Biolegio) (Supporting Information, Table S2), iTag Universal SYBR Green Supermix (Bio‐Rad, 172‐5125), and a QuantStudio 7 Flex (Thermo Fisher).

### 2.7 Flow cytometry

Primary microglia were harvested using Accutase (Sigma‐Aldrich, A6964) and resuspended in medium (HBSS without phenol red (Gibco, 14175‐053), supplemented with 15 mM HEPES, 0.6% glucose, and 1 mM EDTA (Invitrogen, 15575‐020)) after centrifugation. Cells were incubated 15 min on ice in anti‐Cd16/32 Fc blocking solution and subsequently stained with PE‐conjugated anti‐VISTA (clone MIH63, Biolegend, 150204) 30 min on RT. After washing, cells were analyzed on a MacsQuant (Miltenyi Biotec) flow cytometry analyzer. Dapi was used to distinguish dead cells.

### 2.8 Transcription factor motif enrichment analysis

To determine potential TF binding motifs in ATAC‐seq peaks, a motif enrichment analysis was performed using the motif discovery software HOMER (version 4.9) (Salk Institute and University of California San Diego) (Heinz et al., 2010). Peaks on the VISTA locus (eight in total) were identified in Integrative Genomics Viewer (Broad Institute and University of California) and sequences were used for HOMER enrichment analysis (findMotifs, homer2). HOMER analysis determines motifs enriched in sequences compared to scrambled sequences.

### 2.9 Statistical analysis

Statistical analyses were performed using GraphPad Prism 7 (GraphPad Software, Inc.). For multiple comparison after LPS and TLR experiments, a one‐way ANOVA including Dunnett's test for multiple comparison was used. For comparison of gene expression in LPS‐injected mice and *Ercc1* ^*Δ/−*^, a (un)paired Student's *t* test was performed. All error bars indicate mean ± standard deviation (*SD*).

## 3 RESULTS

### 3.1 VISTA is primarily expressed by microglia in human and mouse CNS

Expression of most NCRs (CTLA4, PD‐1, PD‐L1, and more), but not VISTA, has been reported in the CNS by microglia, endothelial cells, astrocytes, oligodendrocytes, and/or neurons (Yshii et al., 2017). Using a combination of immunohistochemistry and flow cytometry, we assessed VISTA expression in mouse and human brain.

**Figure 1:**
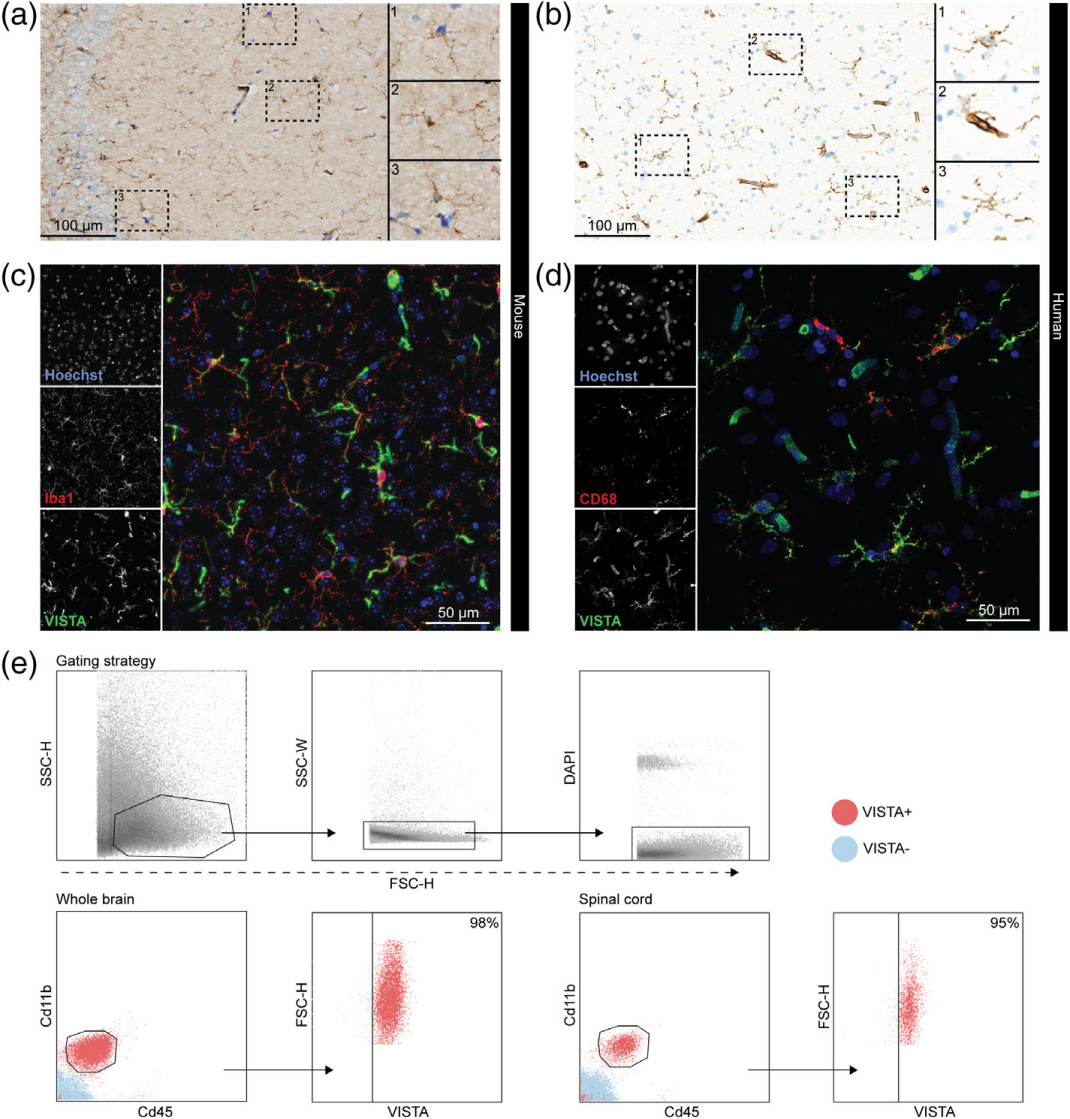
VISTA is expressed by microglia in mouse and human CNS. (a,b) Representative images of VISTA immunoreactivity in mouse (a) (*n* = 2) and human brain (b) (*n* = 4). Inserts show a 2× magnification of areas (1–3) as indicated. (c,d) Representative images of co‐expression of Iba1 (red) and VISTA (green) in mouse brain (c) (*n* = 2) and CD68 (red) and VISTA (green) in human brain (d) (*n* = 4). (e) Representative flow cytometry results of VISTA cell surface expression in Cd11b^+^ Cd45^int^ microglia from brain (*n* = 4) and spinal cord (*n* = 1) [Color figure can be viewed at wileyonlinelibrary.com]

In both mouse and human brain tissue without apparent CNS pathology, VISTA immunoreactivity was evident on ramified microglia‐like cells and on blood vessel structures (Figure 1a,b). Immunofluorescence co‐staining of Iba1 and VISTA (mouse) and CD68 and VISTA (human) revealed a strong co‐expression of these proteins (Figure 1c,d), confirming VISTA expression in microglia.

In accordance with the immunostainings, flow cytometry of whole mouse brain and spinal cord showed that >95% of Cd11b^+^ Cd45^int^ microglia exhibited surface VISTA expression. Furthermore, the vast majority of Cd11b^−^ Cd45^−^ cells did not express detectable levels of cell surface VISTA (Figure 1e).

These findings demonstrate that VISTA is primarily expressed by microglia, and to a lesser extent in blood vessels in the CNS.

### 3.2 VISTA expression is abundant in adult microglia and expression levels are similar to microglia signature genes

**Figure 2:**
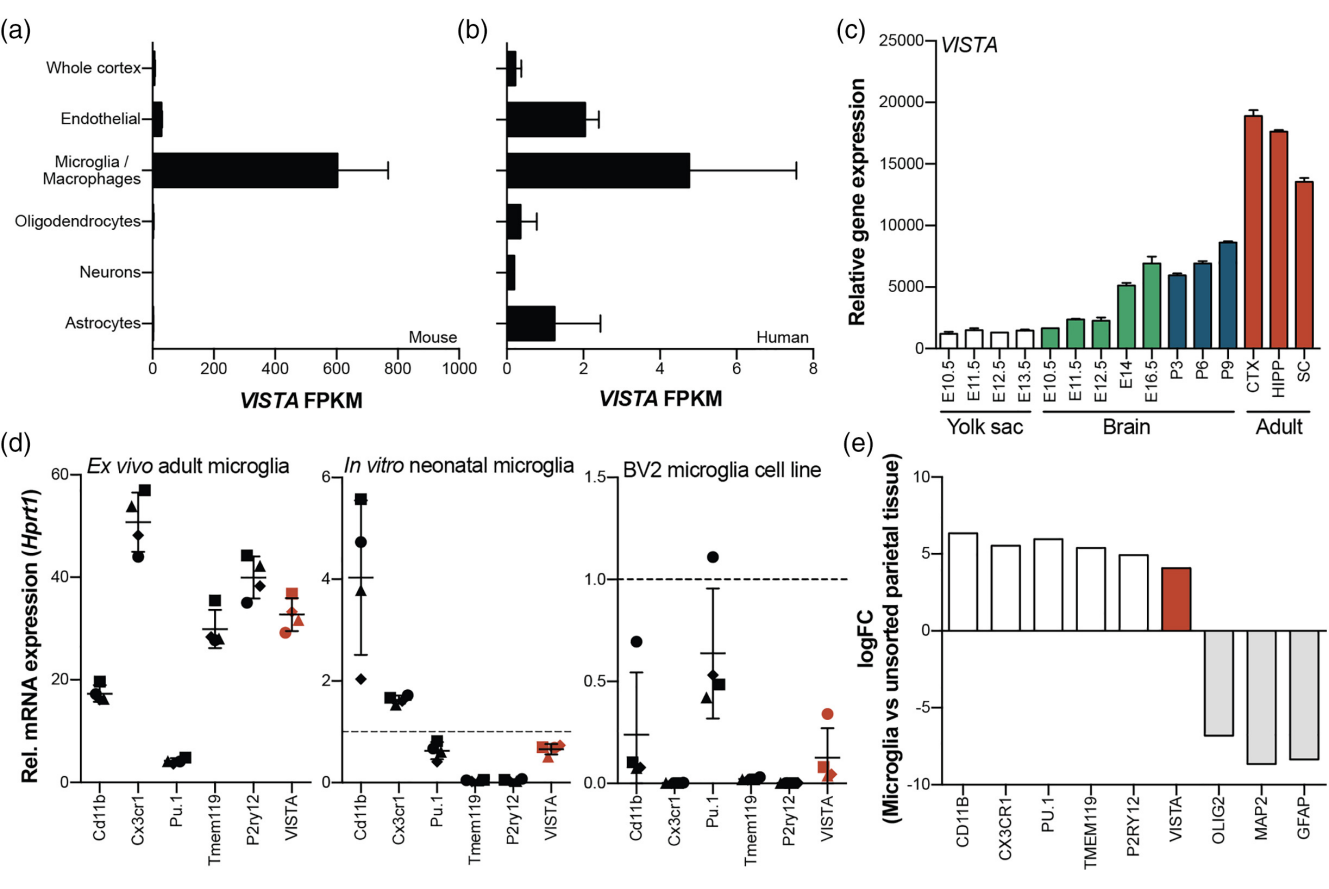
*VISTA* expression is abundant in adult microglia and levels are similar to microglia signature genes. (a,b) *VISTA* mRNA levels (FPKM) in different CNS cell types of mouse (a) and human (b), derived from published RNA‐seq data (Zhang et al., 2014, 2016). (c) Relative gene expression levels of VISTA during microglia development in embryonic yolk sac, brain, and during postnatal development until adulthood, derived from published RNA‐seq data (Matcovitch‐Natan et al., 2016). (d) Relative mRNA expression levels of *VISTA* in sorted adult microglia, cultured primary neonatal microglia, and BV2 cells, measured using RT‐qPCR and normalized to *Hprt1*. (e) LogFC of *VISTA* enrichment in sorted human microglia (*n* = 39) compared to unsorted cortical tissue (*n* = 16), derived from published RNA‐seq data (Galatro et al., 2017a). Error bars indicate mean ± *SD*. FPKM = fragments per kilobase per million [Color figure can be viewed at wileyonlinelibrary.com]

To confirm our observations of VISTA expression in microglia and blood vessels, we analyzed published RNA‐seq data for *VISTA* expression in different CNS cell types (Zhang et al., 2014, 2016). In mouse brain, *VISTA* was abundantly expressed by microglia, weakly expressed by endothelial cells, and was not detected in oligodendrocytes, neurons, and astrocytes (Figure 2a**)**. In human brain, *VISTA* was expressed by microglia, but also at moderate levels by endothelial cells, and at low levels by astrocytes (Figure 2b). *VISTA* expression was very low in oligodendrocytes and neurons. Of note, we did not observe any VISTA immunoreactivity in astrocytes (Figure 1a,b). Expression by endothelial cells is in line with our previous findings of VISTA expression in blood vessels.

Next, we investigated a published RNA‐seq dataset to determine *VISTA* expression in isolated mouse microglia during embryonic and postnatal development (Matcovitch‐Natan et al., 2016). The analysis revealed that *VISTA* expression increases during microglia development (Figure 2c) and was most abundant in adult microglia, independent of the CNS region (hippocampus, cortex, and spinal cord) (Figure 2c).

To further quantify *VISTA* expression in adult microglia, we compared *VISTA* mRNA levels to the expression of microglia signature genes. In acutely isolated adult microglia from mouse brain, *VISTA* was abundantly expressed, similar to levels of *Tmem119* and *P2ry12*, and other microglia signature genes (Figure 2d). In cultured neonatal mouse microglia and the BV2 microglial cell line, *VISTA* expression was moderate, comparable to expression of *Pu.1* (neonatal microglia) or *Cd11b* (BV2) (Figure 2d). For quantification of *VISTA* in human microglia, we analyzed our published RNA‐seq dataset of microglia isolated from adult nondiseased human postmortem brain (Galatro et al., 2017a). *VISTA* expression was enriched in microglia compared to unsorted parietal cortex tissue (logFC 4.1), an enrichment comparable to what was observed for microglia signature genes *TMEM119*, *P2RY12*, *PU.1*, and *CD11B* (Figure 2e).

In summary, these data demonstrate that VISTA is abundantly expressed by adult human and mouse microglia, and that expression is comparable to microglia signature genes.

### 3.3 TLR stimulation of primary neonatal mouse and adult rhesus macaque microglia in vitro leads to downregulation of VISTA expression

Previous studies have reported an upregulation of several NCRs (PD‐1, PD‐L1, and PD‐L2) in microglia and other CNS cells during inflammatory conditions, suggesting a role for NCRs in CNS inflammation. To investigate the expression changes of VISTA during inflammation, we stimulated neonatal mouse microglia and adult rhesus macaque microglia with various TLR agonists in vitro and determined changes in *VISTA* mRNA levels.

**Figure 3:**
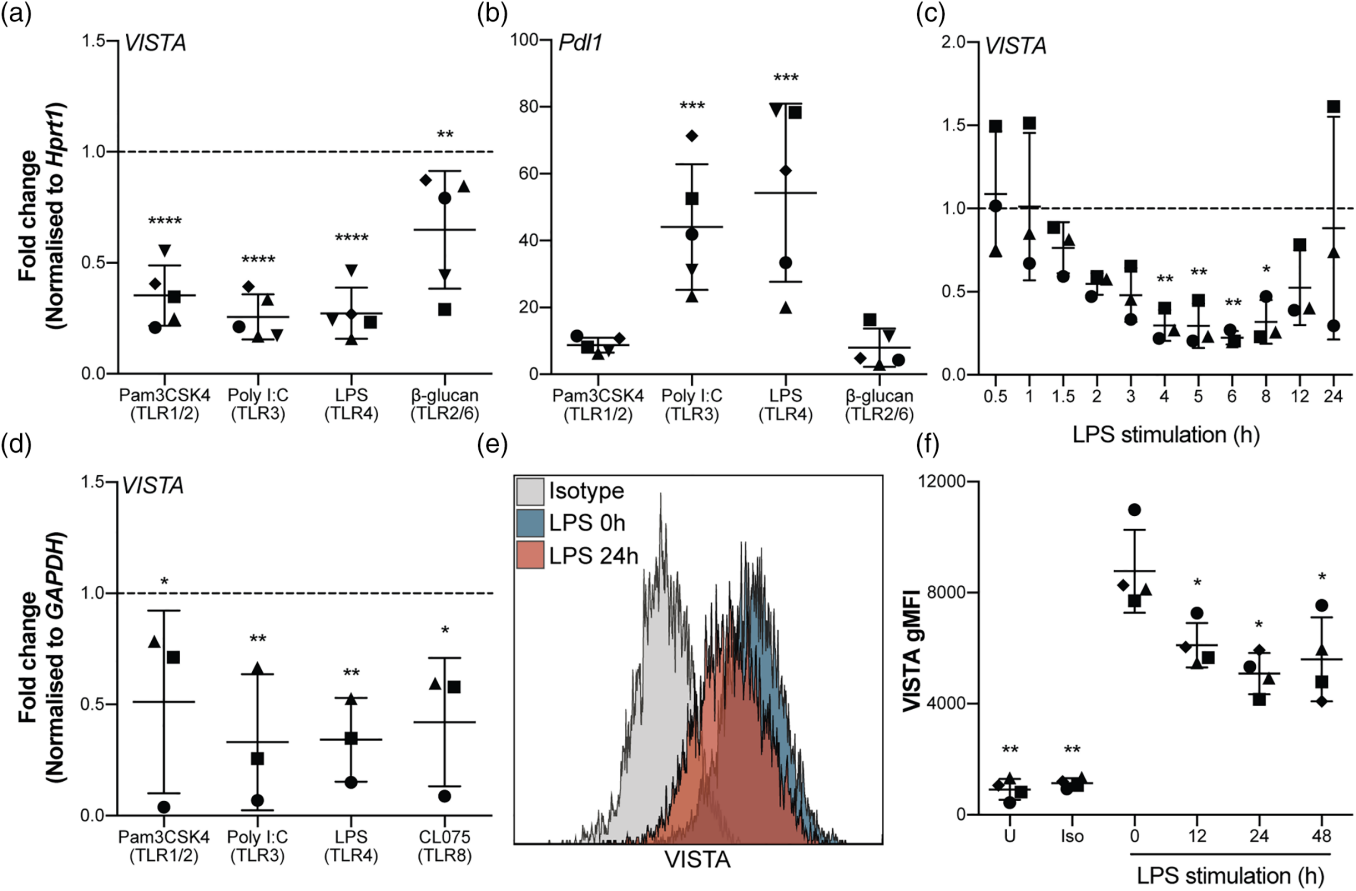
TLR stimulation leads to decreased VISTA expression in primary neonatal mouse and adult macaque microglia in vitro. (a,b) Fold change in *VISTA* (a) and *Pdl1* (b) mRNA in primary neonatal mouse microglia measured using RT‐qPCR after 4 hr stimulation with Pam3CSK4 (TLR1/2), PolyI:C (TLR3), LPS (TLR4), and β‐glucan (TLR2/6) compared to untreated control (*n* = 5). (c) Fold change in *VISTA* mRNA in primary neonatal mouse microglia after LPS (TLR4) stimulation over time (0–24 hr) compared to untreated control (*n* = 3). (d) Fold change in *VISTA* mRNA in primary adult rhesus macaque microglia after 16 hr stimulation with Pam3CSK4 (TLR1/2), PolyI:C (TLR3), LPS (TLR4), and CL075 (TLR8) compared to untreated control (*n* = 3). (e) Representative flow cytometry histogram showing VISTA cell surface expression in primary neonatal mouse microglia after 0 and 24 hr LPS stimulation. (f) Geometric mean fluorescence intensity (gMFI) of VISTA in primary neonatal mouse microglia upon (0, 12, 24, and 48 hr) LPS stimulation (*n* = 4). Statistical analysis conducted was a one‐way ANOVA with Dunnett's test for multiple comparisons. Error bars indicate mean ± *SD*. *p < .05, **p < .01, ***p < .001, ****p < .0001 [Color figure can be viewed at wileyonlinelibrary.com]

After stimulation of TLR1/2, 3, 4, and 2/6, *VISTA* expression was significantly decreased in neonatal mouse microglia by up to 70% (Figure 3a). In contrast, *Pdl1* expression was increased as described in literature (Yshii et al., 2017) (Figure 3b). *VISTA* mRNA decreased as early as 1–2 hr after LPS stimulation and remained reduced up to 12 hr post‐LPS exposure (Figure 3c). Consistently, stimulation of TLR1/2, 3, 4, and 8 in adult rhesus macaque microglia in vitro also decreased *VISTA* expression (Figure 3d).

To show that VISTA surface protein is downregulated as well upon TLR stimulation, flow cytometry on in vitro LPS‐stimulated primary neonatal mouse microglia was performed. VISTA expression was significantly reduced 12–48 hr after LPS stimulation (Figure 3e,f). Whereas the number of VISTA positive microglia decreased only mildly (~10% at 24 hr post‐LPS) (Figure 3e), the geometric mean fluorescence intensity (gMFI), which reflects surface VISTA levels per cell, was strongly reduced (~50% at 24 hr post‐LPS) (Figure 3f).

Following all TLR stimulations, *Tnfα* expression was induced in microglia, demonstrating their immune activation in this experimental setup (Supporting Information, Figure S1).

Our experiments show that VISTA expression is decreased in microglia in response to a range of TLR agonists, which is in contrast to the increased expression of PD‐L1 and other NCRs (Yshii et al., 2017).

### 3.4 *VISTA* expression is reduced upon microglial activation in vivo

In view of the reduced VISTA expression after TLR stimulation of microglia in vitro, we next investigated changes in VISTA expression after microglial activation and inflammation in vivo.

**Figure 4:**
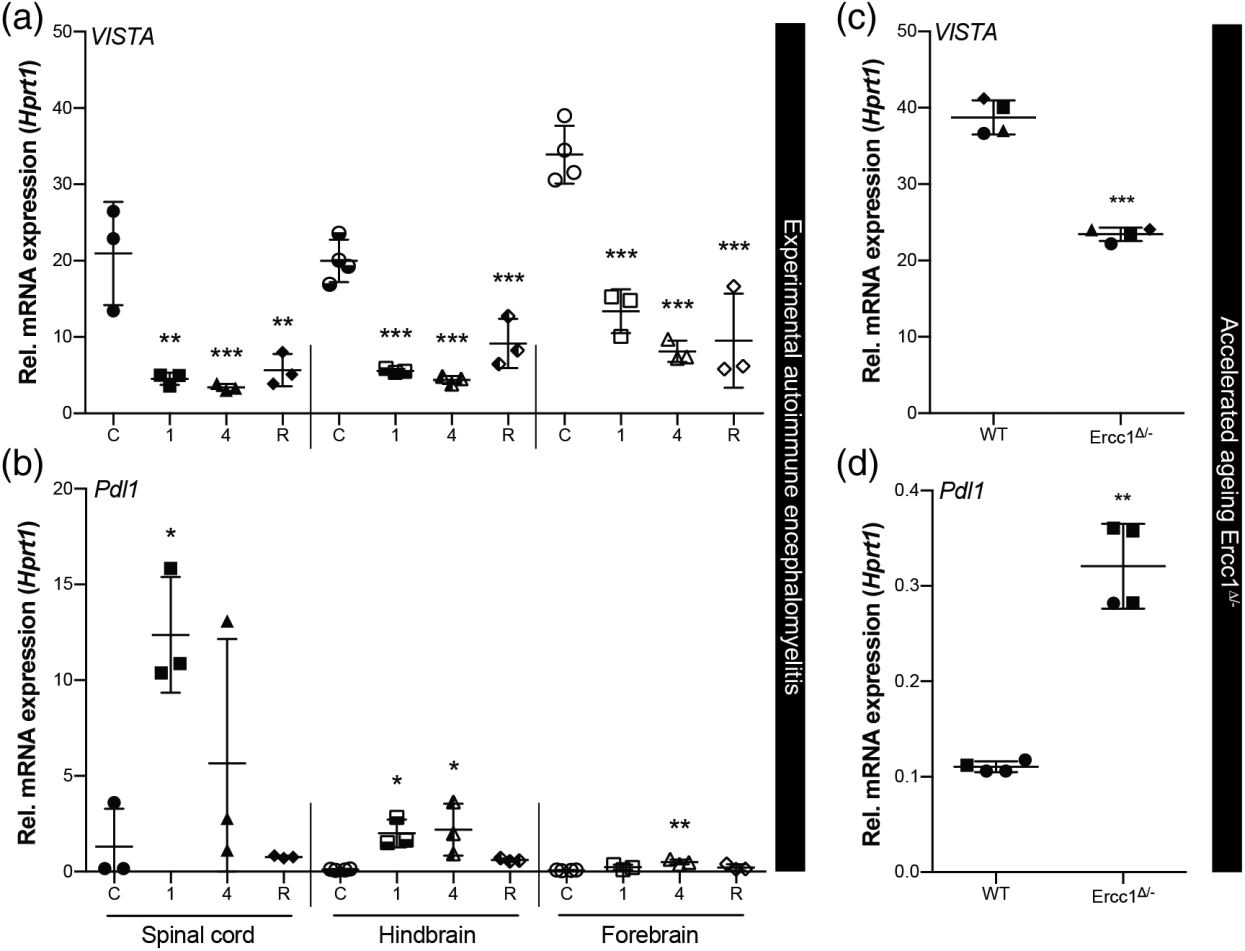
*VISTA* expression is reduced in adult microglia in mouse models of CNS pathology. *VISTA* (a + c) and *Pdl1* (b + d) mRNA levels in acutely isolated adult microglia from spinal cord, hindbrain and forebrain during EAE disease course (C = control, 1 = disease score 1, 4 = disease score 4, R = remission) (a + b), and from whole brain of accelerated aging *Ercc1* ^*Δ/−*^ mice and WT littermates (c + d), measured using RT‐qPCR and normalized to *Hprt1*. Statistical analysis conducted was a one‐way ANOVA with Dunnett's test for multiple comparisons (a + b) and a paired Student's *t* test for direct comparisons (c + d). Error bars indicate mean ± *SD*. WT = wild type, *p < .05, **p < .01, ***p < .001

To address changes in microglia VISTA expression during CNS inflammation, we isolated microglia from mouse CNS at different stages of EAE (induced by MOG_35–55_ in CFA), which is a mouse model of MS. During acute stages of EAE (disease score 1 and 4) microglia obtain a weak immune‐activated phenotype (Vainchtein et al., 2014), indicated by increased levels of *Il1β* (Supporting Information, Figure S2a). In remission, increased *Axl* and MHC‐II component *H2Aa* expression suggest a phagocytic and antigen‐presenting phenotype of microglia (Supporting Information, Figure S2a). We observed a significant decrease in *VISTA* expression in all stages of EAE (score 1, 4, and remission) in spinal cord, hindbrain, and forebrain microglia compared to nonimmunized mice (Figure 4a). In contrast, *Pdl1* was upregulated in all conditions (Figure 4b**)**.

To further assess VISTA expression changes during microglial activation, we quantified *VISTA* mRNA in microglia isolated from *Ercc1* ^*Δ/−*^ mice. Ercc1 is a protein essential for nucleotide excision DNA repair and mutant mice display an accelerated aging phenotype (Vermeij et al., 2016). Microglia from whole brain of 4‐month‐old *Ercc1* ^*Δ/−*^ mice exhibited increased *Il1β* and *Axl* expression (Supporting Information, Figure S2b), indicating an immune‐activated and phagocytic phenotype. *VISTA* expression in these microglia was significantly reduced compared to wild type (WT) littermates (Figure 4c), whereas *Pdl1* expression was increased (Figure 4d).

These data demonstrate that VISTA expression is decreased in microglia in different mouse models of CNS inflammation and during microglial activation, which is in line with our in vitro observations.

### 3.5 Reduced VISTA expression in LPS‐activated microglia is accompanied by chromatin remodeling

To determine if changes in *VISTA* expression are accompanied by epigenetic alterations, we analyzed a recently generated dataset containing genome‐wide transcriptional changes (RNA‐seq), histone modifications (ChIP‐seq), and chromatin accessibility (ATAC‐seq) in isolated microglia after LPS exposure in mice (Zhang et al., in press).

**Figure 5:**
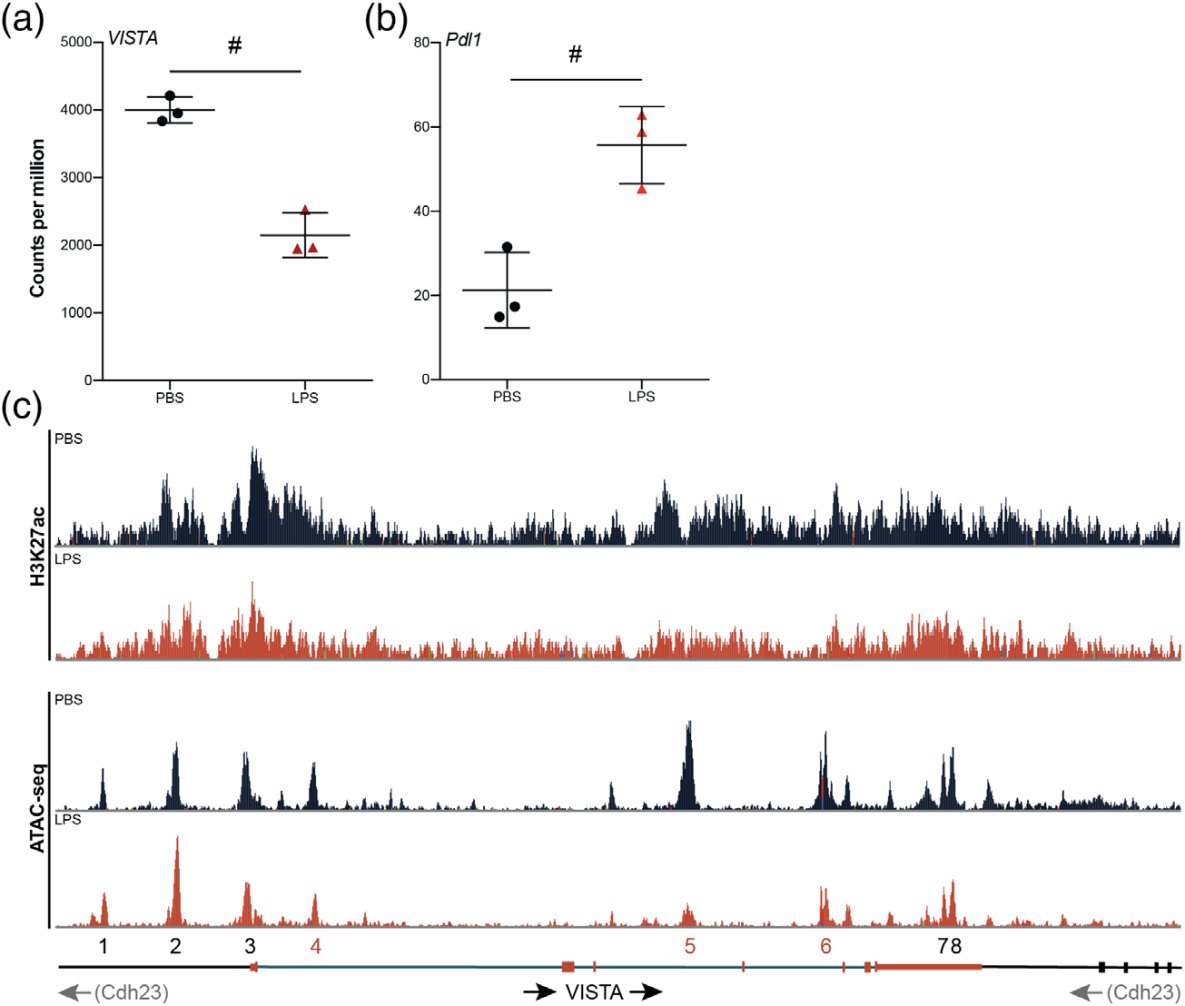
Decreased *VISTA* expression after LPS injection is associated with chromatin remodeling in microglia. (a,b) RNA‐seq counts per million of *VISTA* (a) and *Pdl1* (b) mRNA expression (*n* = 3). (c) H3K27ac histone acetylation (top) and ATAC‐seq (bottom) peaks corresponding to the *VISTA* gene (*n* = 3). ATAC‐seq peaks were numbered 1–8 and peaks that were decreased after LPS stimulation are indicated in red (4–6). Data are derived from previously generated datasets (Zhang et al., in press). Transcription factor binding motifs enriched in these peaks are listed in Table 1. Error bars indicate mean ± *SD*. # = differential expression (DE) based on RNA‐seq analysis [Color figure can be viewed at wileyonlinelibrary.com]

RNA‐seq data indicated that *VISTA* expression is reduced in acutely isolated microglia 3 hr after LPS injection (Figure 5a), whereas *Pdl1* expression was upregulated (Figure 5b). These results are in line with our previous observations of decreased *VISTA* expression in microglia during EAE and in *Ercc1* ^*Δ/−*^ mice. *VISTA* is located within an intronic region of *CDH23*, a gene associated with auditory function (Johnson et al., 2017). *Cdh23* was not altered in response to LPS, demonstrating that changes in *VISTA* expression were independent of the *Cdh23* gene (Supporting Information, Figure S3a).

To determine if decreased *VISTA* expression is accompanied by epigenetic changes in the gene locus, we analysed ChIP‐seq and ATAC‐seq datasets. Concomitant with reduced *VISTA* expression after LPS exposure, we observed decreased H3K27 histone acetylation (H3K27ac) upstream of the *VISTA* gene (Figure 5c). H3K27ac is enriched on enhancers and associated with active gene transcription.

We next assessed chromatin accessibility using ATAC‐seq data. ATAC‐seq provides information about transposase‐accessibility of chromatin at specific locations on the genome (Buenrostro, Wu, Chang, & Greenleaf, 2015). Transposase‐accessible chromatin is also accessible for transcription factors (TF) and indicated as Peaks (1–8) on the *VISTA* gene (Figure 5c). Enrichment analysis for putative TF binding motifs revealed that 17 consensus sequences were significantly enriched in the DNA underlying these peaks (Table 1). Consensus binding sites for Pu.1 (Spi1) and Mafb were among the most significantly enriched TF motifs, and both proteins are crucial for microglia function (Matcovitch‐Natan et al., 2016; Smith et al., 2013). In DNA sequences of ATAC peaks that were reduced in microglia after LPS injection, we detected consensus binding sites for Pu.1, Rfx6, Elf5, and Sox15 (Peaks 4–6) (Figure 5c and Table 1). In contrast, Ap4 and Nf1 binding motifs were enriched in DNA sequences of peaks unaltered by LPS stimulation (Peaks 1–3, 7–8) (Figure 5c and Table 1).

**Table 1:** Transcription factor motifs enriched in ATAC‐seq peaks on the *VISTA* gene (ordered from high to low enrichment score) [Color table can be viewed at wileyonlinelibrary.com]

Motif	Consensus	*p* value	*q* value (benjamini)	# Peaks with motif (of 8)
Pu.1	AGAGGAAGTG	1,00E‐05	0.0017	5.0
Rfx6	TGTTKCCTAGCAACM	1,00E‐04	0.0079	7.0
Elf5	ACVAGGAAGT	1,00E‐03	0.0184	5.0
Sox15	RAACAATGGN	1,00E‐03	0.0548	4.0
Mafb	WNTGCTGASTCAGCANWTTY	1,00E‐03	0.0675	3.0
Zfp281	CCCCTCCCCCAC	1,00E‐02	0.1110	2.0
Nr5a2	BTCAAGGTCA	1,00E‐02	0.1110	2.0
Znf143	ATTTCCCAGVAKSCY	1,00E‐02	0.1110	2.0
Klf10	GGGGGTGTGTCC	1,00E‐02	0.1247	3.0
Ap4	NAHCAGCTGD	1,00E‐02	0.1657	4.0
Scl	AVCAGCTG	1,00E‐02	0.2013	8.0
Prdm14	RGGTCTCTAACY	1,00E‐02	0.2013	2.0
Fxr, Ir1	AGGTCANTGACCTB	1,00E‐02	0.2013	2.0
MafK	GCTGASTCAGCA	1,00E‐02	0.2013	2.0
Sox4	YCTTTGTTCC	1,00E‐02	0.2013	4.0
Elf3	ANCAGGAAGT	1,00E‐02	0.2225	3.0
Nf1	YTGCCAAG	1,00E‐02	0.2225	6.0

Motifs enriched in peaks decreased upon LPS (Peaks 4–6).

Motifs enriched in peaks unchanged upon LPS (Peaks 1–3, 7, and 8).

Motifs enriched in both unchanged peaks and peaks decreased by LPS.

Our findings show that reduced *VISTA* expression is accompanied by altered histone modification enrichments and changes in chromatin accessibility that are associated with transcriptional repression. Furthermore, the presence of consensus binding sites for Pu.1 and Mafb on chromatin accessible DNA on the *VISTA* gene suggests that VISTA may be regulated by these microglia‐specific TF, and that reduced accessibility of Pu.1, Elf5, and Sox15 to consensus binding sites in *VISTA* underlie reduced expression in response to LPS.

### 3.6 VISTA expression is differentially regulated in the human CNS

In view of the observed reduction in VISTA expression during microglial activation in vitro and in vivo, we next assessed VISTA expression in human brain tissue of young and old individuals, and in septicemia, MS and AD patients (Supporting Information, Table 3). Based on neuropathological evaluations, one representative patient was selected for analysis of each condition.

**Figure 6:**
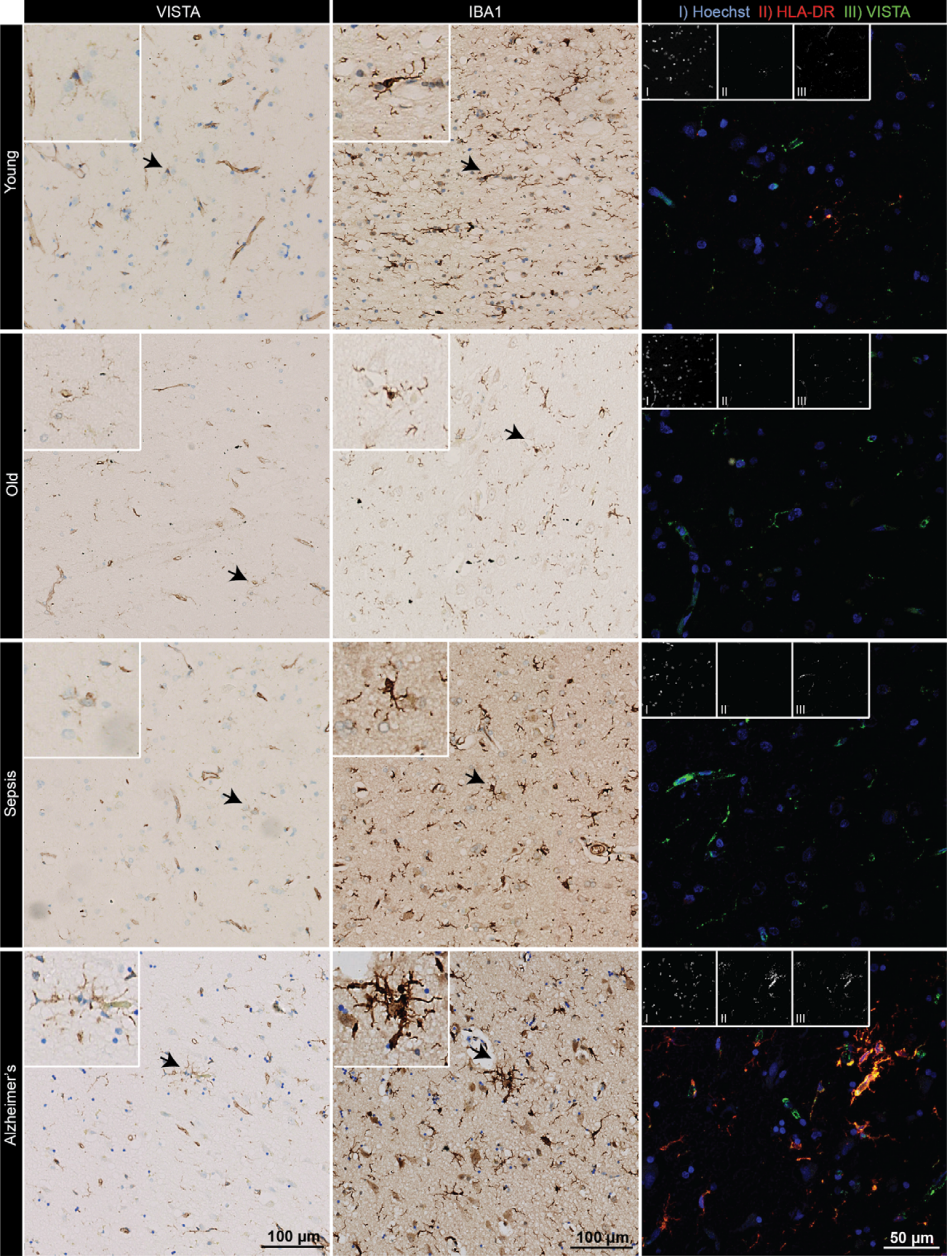
VISTA expression in young and old individuals, and sepsis and Alzheimer's patients. VISTA and IBA1 immunoreactivity, and immunofluorescence of HLA‐DR (red) and VISTA (green) co‐expression in human brain tissue of young, old, sepsis, and AD patients (*n* = 1) [Color figure can be viewed at wileyonlinelibrary.com]

**Figure 7:**
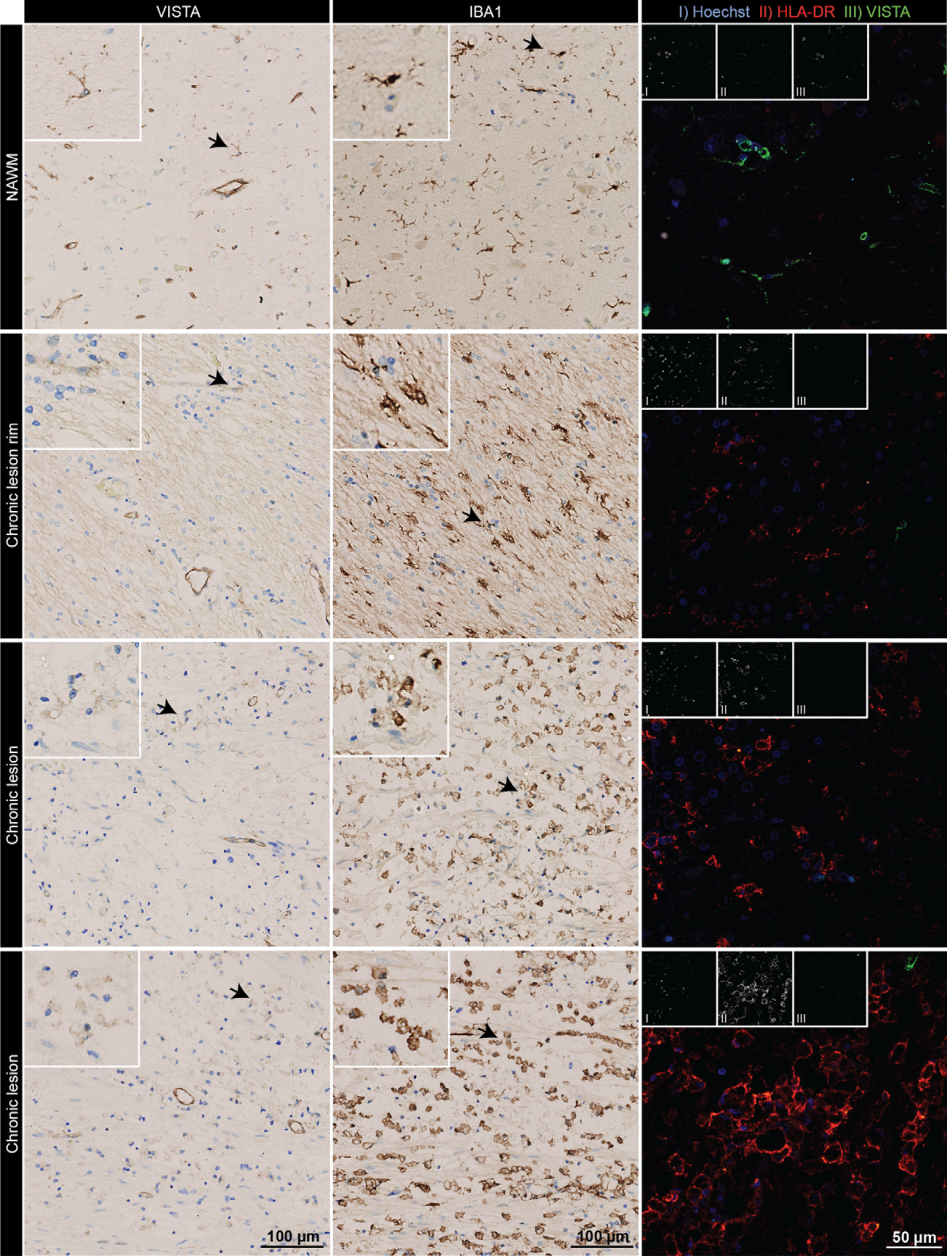
VISTA expression is reduced in and around chronic MS lesions. VISTA and IBA1 immunoreactivity, and immunofluorescence of HLA‐DR (red) and VISTA (green) co‐expression in different lesion regions (Van Der Valk & De Groot, 2000) of an MS patient (*n* = 1). NAWM = normal appearing white matter [Color figure can be viewed at wileyonlinelibrary.com]

Using IBA1 immunostaining, microglial activation was assessed based on morphology and staining intensity, and VISTA immunoreactivity was determined in consecutive tissue sections (Figures 6 and 7). In tissue of a young individual (27 years), microglia exhibited a typical resting, ramified morphology, and VISTA expression was detected on microglia and endothelial cells (Figure 6). In both the old individual (70 years) and the septicemia patient, we observed only weakly activated microglia, and VISTA immunoreactivity in microglia was slightly reduced (Figure 6 and Table 2). VISTA staining of endothelium, however, did not seem to be affected. In the AD patient, a strong IBA1 staining intensity suggested microglial activation, which correlated with strong VISTA expression, and was specifically observed in microglia clusters (Figure 6). Co‐staining of IBA1 and β‐amyloid revealed that these microglia clusters were surrounding β‐amyloid plaque (Supporting Information, Figure S4). No difference in endothelial VISTA expression was observed. In MS normal‐appearing white matter (NAWM), microglial activation was low and VISTA was highly expressed on microglia and endothelium (Figure 7 and Table 2). Within and around a chronic lesion in the MS tissue, intermediate to strong IBA1 staining was observed, whereas VISTA staining was almost absent in microglia and endothelial cells.

**Table 2:** Summary of VISTA immunoreactivity in human CNS pathologies

Pathology	Immunohistochemistry			Immunofluorescence	
Aging, sepsis, AD	Microglial activation^a^	VISTA microglia	VISTA endothelium	HLA‐DR	VISTA
Young	−	+	+	−	+
Old	++	+/−	+	+/−	+
Sepsis	+	+/−	+	+/−	+
AD	+	++	+	++	++
Multiple sclerosis					
NAWM	−	++	++	+	++
Chronic lesion rim	+/−	−	+	+	+
Chronic lesion 1	++	−	+/−	++	−
Chronic lesion 2	++	+/−	−	++	+/−

*Note*. NAWM = normal appearing white matter.

++, high expression/activation; +, normal expression/activation; +/−, weak expression/activation; −, no expression/activation (expression and activation scored relative to young individual; i.e., normal VISTA expression and no microglial activation).

a Microglia activation based on IBA1 staining and morphology compared to young individual.

To establish a correlation between VISTA expression and CNS inflammation in these tissues, we performed a co‐staining of VISTA with HLA‐DR (Figures 6 and 7). In line with the previous immunostaining, low levels of HLA‐DR in the young individual, and positive VISTA signals in microglia and endothelial cells were observed (Figure 6 and Table 2). In the old and the septicemia donors, HLA‐DR expression was only slightly increased, but VISTA expression remained unchanged. In the AD tissue, HLA‐DR was highly expressed by microglia, which also abundantly expressed VISTA. In MS, co‐staining revealed a negative correlation of HLA‐DR and VISTA expression (Figure 7 and Table 2). VISTA was not detected in microglia/macrophages and endothelial cells at the rim of the lesion; however, a weak VISTA signal was observed in some amoeboid cells expressing high HLA‐DR within the lesion.

Our in situ analyses of human specimens indicate that regulation of VISTA expression during CNS pathology is more variable in human disease. In line with our mouse data (Figure 4a), we observed reduced VISTA expression in MS. Conversely, in AD, VISTA expression positively correlated with microglial activation.

## 4 DISCUSSION

In this article, we demonstrate that VISTA is expressed by mouse and human microglia and endothelial cells in the CNS, and that expression is differentially regulated during disease. In view of increasing interest for the role of NCRs in the CNS, and the unexpected adverse neurological effects following immunotherapy, we provide first evidence for a possible function of VISTA in microglia and during CNS pathology. Key findings include the following: (a) VISTA is abundantly expressed by human and mouse microglia comparable to microglia signature genes. (b) *VISTA* expression is downregulated in mouse and rhesus macaque microglia upon TLR ligation in vitro*,* and in mouse microglia during EAE, accelerated aging, and by LPS stimulation. (c) Our results suggest that *VISTA* expression is potentially regulated by the two microglia‐specific TF, Pu.1, and Mafb, and that reduced *VISTA* expression after LPS injection is associated with histone modifications and reduced chromatin accessibility. (d) Finally, our findings indicate that VISTA expression is differentially regulated in human CNS pathologies including MS and AD.

In mouse and human brain, VISTA mRNA and protein were primarily detected in microglia. Surface VISTA was detected on >95% of microglia in mice, and gene expression in mouse and human microglia was similar to levels of microglia signature genes. Hence, VISTA is abundantly expressed by microglia. Regarding VISTA function in T cell inhibition, it is surprising that expression is high in microglia under nonpathological conditions, as T cells are not present in healthy brain parenchyma. However, it is possible that VISTA expression by microglia is necessary to assure T cell suppression during immunosurveillance of the CNS (Korn & Kallies, 2017), or to limit tissue damage in case of T cell infiltration under pathological conditions. In addition, other functions of VISTA have been reported, which could play a role in microglia homeostasis and innate immune response. For example, Yoon et al. (2015) showed that VISTA expression in phagocytic cells is essential for uptake of apoptotic cells. Furthermore, VISTA is involved in the immune response of myeloid cells, as overexpression in human monocytes leads to spontaneous cytokine secretion (Bharaj et al., 2014). Thus, VISTA may be involved in immune surveillance and uptake of apoptotic neurons or other debris by microglia.

Consistent with this argument, our findings indicate that VISTA expression is regulated similarly to known homeostatic microglia genes such as TMEM119 and P2RY12. These signature genes are downregulated upon microglial activation (Grabert et al., 2016). Expression of P2RY12 is lost in active MS lesions (Zrzavy et al., 2017), in which microglia obtain an immune‐activated phenotype. The decrease in VISTA expression during microglial activation in mice and in MS lesions indicates a similar homeostatic function of VISTA. Supporting this notion, we identified consensus binding sites for Pu.1 and Mafb in accessible chromatin on the VISTA gene, which are TFs pivotal for microglia homeostatic function. Knockout of Pu.1 in mice leads to a complete loss of microglia (Smith et al., 2013), and Mafb is involved in homeostasis of adult microglia (Matcovitch‐Natan et al., 2016).

The downregulation of VISTA that we observed in activated microglia stands in marked contrast to published studies on expression of other NCRs (Yshii et al., 2017). In microglia, expression of several NCRs is induced or upregulated upon inflammatory stimuli (Yshii et al., 2017), which we confirmed for PD‐L1 expression in this study. Moreover, VISTA expression is upregulated in TLR‐stimulated monocytes and macrophages (Bharaj et al., 2014; Wang et al., 2011). This discrepancy underscores our previous argument that VISTA has additional functions in microglia that deviate from other NCRs and other myeloid cells. Considering the function of VISTA in apoptotic cell clearance and cytokine response (Bharaj et al., 2014; Yoon et al., 2015), a loss of VISTA in activated microglia may reduce their ability to clear debris or to mount a cytokine response. However, VISTA may also function as an NCR in microglia, and downregulation likely has consequences for CNS pathologies which involve T cell infiltration. Several studies have shown that knockout of NCRs including VISTA in mice promotes the development of EAE (Joller et al., 2012; Wang et al., 2014). Hence, lack of VISTA expression on microglia may promote T cell infiltration and activation in the CNS, which can exacerbate or predispose for diseases such as MS or EAE. The function of VISTA in microglia with regard to CNS pathology should be evaluated in further studies.

Our results also indicate a potential underlying epigenetic mechanism by which *VISTA* expression is reduced upon LPS stimulation in microglia. We observed decreased H3K27ac enrichment, a histone modification associated with active transcription. Furthermore, Pu.1, Rfx6, Elf5, and Sox15 consensus binding motifs in the *VISTA* gene exhibited reduced accessibility after LPS stimulation. Together, these epigenetic alterations likely contribute to reduced expression of VISTA in LPS‐activated microglia.

In line with our findings of decreased VISTA expression in activated microglia, we observed a strong reduction of VISTA on microglia/macrophages in chronic MS lesion tissue. Strikingly, VISTA expression was elevated in microglia in NAWM and close to plaques in the AD patient compared to the young and old individual. Therefore, expression regulation of VISTA in human CNS disease could depend on the underlying pathophysiology. In chronic MS lesions, massive infiltration and unregulated activity of macrophages and lymphocytes occur (Van Der Valk & De Groot, 2000). It is possible that the presence of immune cell infiltrates in MS might facilitate the loss of VISTA expression, and VISTA deficiency could again augment infiltration. In contrast, AD is a neurodegenerative disease which features endogenous inflammation with absence of parenchymal immune cell infiltrates (Graeber, Li, & Rodriguez, 2011). In AD, microglia are activated by amyloid‐β (Aβ) and neuronal debris, contributing to clearance, but also causing tissue damage. A recent study suggests that aggregated Aβ sensed by microglia causes inflammasome activation, which contributes to progression and spreading of inflammation and Aβ pathology (Venegas et al., 2017). Furthermore, single‐cell RNA‐sequence data suggest that plaque‐associated microglia in mice obtain a Trem2‐dependent phagocytic phenotype (Keren‐Shaul et al., 2017). Hence, VISTA expression changes in activated microglia may depend on environmental cues in CNS pathologies, such as interactions with peripheral immune infiltrates in MS, or activation by Aβ in AD.

Interestingly, we also observed downregulation of VISTA in endothelial cells in chronic MS lesion. The endothelium is involved in MS pathology by recruitment of immune cells to the CNS (Yun, Minagar, & Alexander, 2017). Endothelial cells are involved in antigen presentation of CNS components to antigen‐specific lymphocytes (Galea et al., 2007; Lopes Pinheiro et al., 2016; Traugott & Raine, 1985). Blocking co‐inhibitory molecules PD‐L1 or PD‐L2 on human endothelial cells facilitates transmigration responses of lymphocytes in vitro (Pittet, Newcombe, Prat, & Arbour, 2011; Rodig et al., 2003). Reduced VISTA expression on endothelium in MS could therefore promote activation and transmigration of lymphocytes into the CNS. Additional studies are needed to assess the effect of VISTA deficiency in endothelial cells regarding antigen presentation and immune cell infiltration.

In conclusion, we present an elaborate multi‐species analysis of VISTA expression in the CNS, including changes during pathology. We demonstrate that VISTA is abundantly expressed by microglia, suggesting a functional role in these cells. Differential expression of VISTA during CNS pathology highlights the importance to further elucidate the function of VISTA in the CNS. Our study is the first to show VISTA expression patterns in the CNS, which serves as a basis for future studies to address the role of VISTA in microglia and during CNS pathology.

## CONFLICTS OF INTEREST

Dr Randolph J. Noelle is CSO of ImmuNext Inc., and is involved with the commercial development of VISTA.

## Supporting information


**Supporting Information, Table 1** Primary antibodies used for immunohistochemical labeling
**Supporting Information, Table 2**. Primers used for quantitative real‐time PCR
**Supporting Information, Table 3**. Patient information
**Supporting Information, Figure 1**. TLR stimulation drives *Tnfα* expression in primary neonatal mouse microglia in vitro. (a,b) LogFC of *Tnfα* gene expression measured using RT‐qPCR after stimulation with different TLR agonists (a) (*n* = 5) and LPS over time (b) compared to untreated control (*n* = 3). Statistical analysis conducted was a one‐way ANOVA with Dunnett's test for multiple comparisons. Error bars indicate mean ± *SD*. ***p < .001, ****p < .0001
**Supporting Information, Figure 2**. Acutely isolated adult microglia from Ercc1^Δ/−^ and EAE mice exhibit an immune‐activated phenotype. (a) Relative gene expression of *Il1b*, *Axl*, and *H2Aa* in acutely isolated adult microglia from spinal cord (*Il1b, Axl, H2Aa*) and hindbrain (*H2Aa*) from EAE control, score 1, score 4, and remission (*n* = 3). (b) Relative gene expression of *Il1b* and *Axl* in acutely isolated adult microglia from whole brain of *Ercc1* ^*Δ/−*^ mice and WT littermates. Gene expression was measured using RT‐qPCR and data are normalised to Hprt1. Statistical analysis conducted was a one‐way ANOVA with Dunnett's test for multiple comparisons (a) and a paired Student's *t* test (b). Error bars indicate mean ± *SD*. *p < .05, **p < .01, ***p < .001
**Supporting Information, Figure 3**. VISTA H3K4 histone tri‐methylation and *Cdh23* and *Il1β* expression in microglia after LPS injection. (a,b) RNA‐seq counts per million of *Cdh23* (a) and *Il1β* (b) mRNA expression (*n* = 3). (c) H3K4me3 histone tri‐methylation peaks corresponding to the VISTA gene (*n* = 3). Data are derived from previously generated datasets (Zhang et al., in preparation). Error bars indicate mean ± *SD*. # = Differential expression (DE) based on RNA‐sequence analysis
**Supporting Information, Figure 4**. Microglia cluster around β‐amyloid plaque in Alzheimer's patients. Immunofluorescence staining of β‐amyloid (red) and IBA1 (green) in human brain tissue of an Alzheimer's patient (*n* = 1)
