# Supplementary material for: VISTA expression by microglia decreases during inflammation and is differentially regulated in CNS diseases
Source: Glia. 2018 Oct 11;66(12):2645–58. doi: 10.1002/glia.23517 (PMC6585704; doi:10.1002/glia.23517)
Supplement: Supplementary file 1 — Supporting Information, Table 1 Primary antibodies used for immunohistochemical labeling Supporting Information, Table 2. Primers used for quantitative real‐time PCR Supporting Information, Table 3. Patient information Supporting Information, Figure 1. TLR stimulation drives Tnfα expression in primary neonatal mouse microglia in vitro. (a,b) LogFC of Tnfα gene expression measured using RT‐qPCR after stimulation with different TLR agonists (a) (n = 5) and LPS over time (b) compared to untreated control (n = 3). Statistical analysis conducted was a one‐way ANOVA with Dunnett's test for multiple comparisons. Error bars indicate mean ± SD. ***p < .001, ****p < .0001 Supporting Information, Figure 2. Acutely isolated adult microglia from Ercc1Δ/− and EAE mice exhibit an immune‐activated phenotype. (a) Relative gene expression of Il1b, Axl, and H2Aa in acutely isolated adult microglia from spinal cord (Il1b, Axl, H2Aa) and hindbrain (H2Aa) from EAE control, score 1, score 4, and remission (n = 3). (b) Relative gene expression of Il1b and Axl in acutely isolated adult microglia from whole brain of Ercc1 Δ/− mice and WT littermates. Gene expression was measured using RT‐qPCR and data are normalised to Hprt1. Statistical analysis conducted was a one‐way ANOVA with Dunnett's test for multiple comparisons (a) and a paired Student's t test (b). Error bars indicate mean ± SD. *p < .05, **p < .01, ***p < .001 Supporting Information, Figure 3. VISTA H3K4 histone tri‐methylation and Cdh23 and Il1β expression in microglia after LPS injection. (a,b) RNA‐seq counts per million of Cdh23 (a) and Il1β (b) mRNA expression (n = 3). (c) H3K4me3 histone tri‐methylation peaks corresponding to the VISTA gene (n = 3). Data are derived from previously generated datasets (Zhang et al., in preparation). Error bars indicate mean ± SD. # = Differential expression (DE) based on RNA‐sequence analysis Supporting Information, Figure 4. Microglia cluster around β‐amyloid plaque in Alzheimer's patients. I [file GLIA-66-2645-s001.docx]

# **Supporting Information**

**Supplementary Table 1.** Primary antibodies used for immunohistochemical labelling

| *Target* | *Reactivity* | *Clone* | *Manufacturer* | *catalog number* | *Working conc.* | *Antigen retrieval* |
| --- | --- | --- | --- | --- | --- | --- |
| Beta-amyloid | Human | 6F/3D | Dako | M0872 | 1.2 μg/mL | Sodium citrate (pH 6) |
| CD68 | Human | PG-M1 | Dako | M0876 | 0.2 μg/mL | Sodium citrate (pH 6) |
| HLA-DR | Human | LN3 | eBioscience | 14-9956-82 | 0.7 μg/mL | Sodium citrate (pH 6) |
| Iba1 | Human, Mouse | Polyclonal | Wako | 019-19741 | 0.5 μg/mL | Sodium citrate (pH 6) |
| VISTA | Human | D1L2G | Cell signalling | #64953 | 5 μL/mL | Sodium citrate (pH 6) |
| VISTA | Mouse | MH5a | Biolegend | 143702 | 10 μg/mL (IHC)  3 μg/mL (TSA) | Tris-EDTA (pH 9) |

**Supplementary Table 2.** Primers used for quantitative real-time PCR

| *Gene* | *Forward primer* | *Reverse primer* |
| --- | --- | --- |
| Axl | TGAAGCCACCTTGAACAGTC | GCCAAATTCTCCTTCTCCCA |
| Cx3cr1 | CTTGCCTCTGGTGGAGTCTG | GTGAGGTCCTGAGCAGATGG |
| HPRT1 | ATACAGGCCAGACTTTGTTGGA | TGCGCTCATCTTAGGCTTTGTA |
| Il1b | CCCAAAAGATGAAGGGCTGC | TGATACTGCCTGCCTGAAGC |
| Itgam (Cd11b) | AACCATCCCATCTTTCCTGCTAA | TGTTGTTCTCACTGGCCACAAT |
| P2ry12 | CAACTCACCTTCACCGGCA | GCCTTGAGTGTTTCTGTAGGGT |
| Pd-l1 (Cd274) | GATCATCCCAGAACTGCCTG | GACACTACAATGAGGAACAACAG |
| Pu.1 (Spi1) | TACAGCAGCTCTATCGCCAC | GCATGTAGGAAACCTGGTGACT |
| Tmem119 | CTTCACCCAGAGCTGGTTCC | GTGACACAGAGTAGGCCACC |
| Tnfa | TCTTCTGTCTACTGAACTTCGG | AAGATGATCTGAGTGTGAGGG |
| VISTA | AACAACGGTTCTACGGGTCC | CGTGATGCTGTCACTGTCCT |
| GAPDH (Macaque) | CAACGAATTTGGCTACAGCA | GTGGTCCGGGGGTCTTAC |
| VISTA (MACAQUE) | CGGCAGCCTTCTGAGTCT | AGAGTCAGGAACAGGGTCCA |

**Supplementary Table 3.** Patient information

| *patient* | *Brain region* | *pathology* | *sex* | *age* | *cause of death* |
| --- | --- | --- | --- | --- | --- |
| young | Frontal area | No brain pathology | Male | 27 | Arrhythmia |
| old | Frontal area | No brain pathology | Male | 70 | Gastrointestinal bleeding |
| sepsis | Frontal area | No brain pathology | Male | 44 | Septicaemia |
| alzheimer’s disease | Hippocampus & entorhinal cortex | AD (Braak stage V) | Male | 74 | Aortic rupture |
| multiple sclerosis | Corpus callosum | End-stage MS | Female | 61 | End-stage MS |


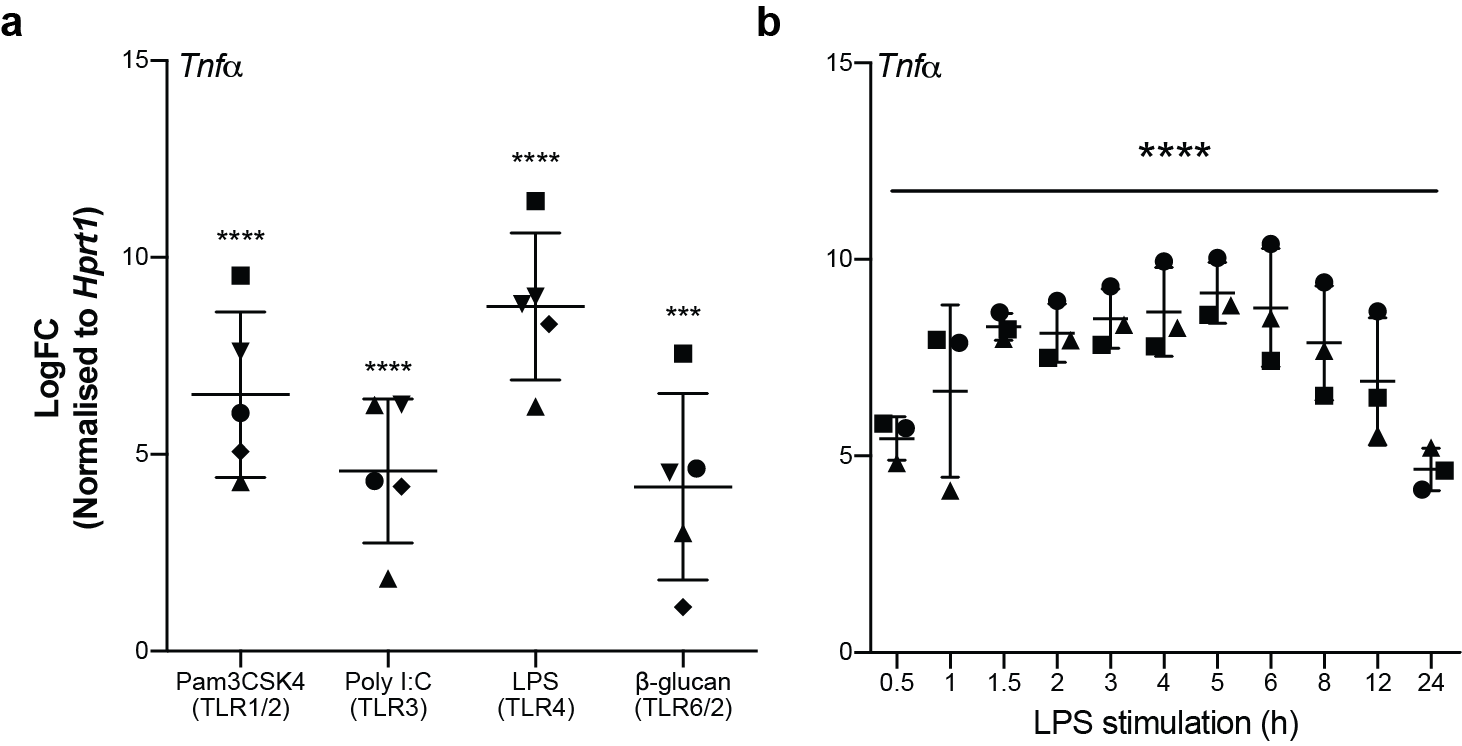


**Supplementary Figure 1.** TLR stimulation drives *Tnfα* expression in primary neonatal mouse microglia *in vitro*. **a-b** LogFC of *Tnfα* gene expression measured using RT-qPCR after stimulation with different TLR agonists (**a**) (n=5) and LPS over time (**b**) compared to untreated control (n=3). Statistical analysis conducted was a one-way ANOVA with Dunnett’s test for multiple comparisons. Error bars indicate mean ± s.d. ***p < 0.001, ****p < 0.0001.


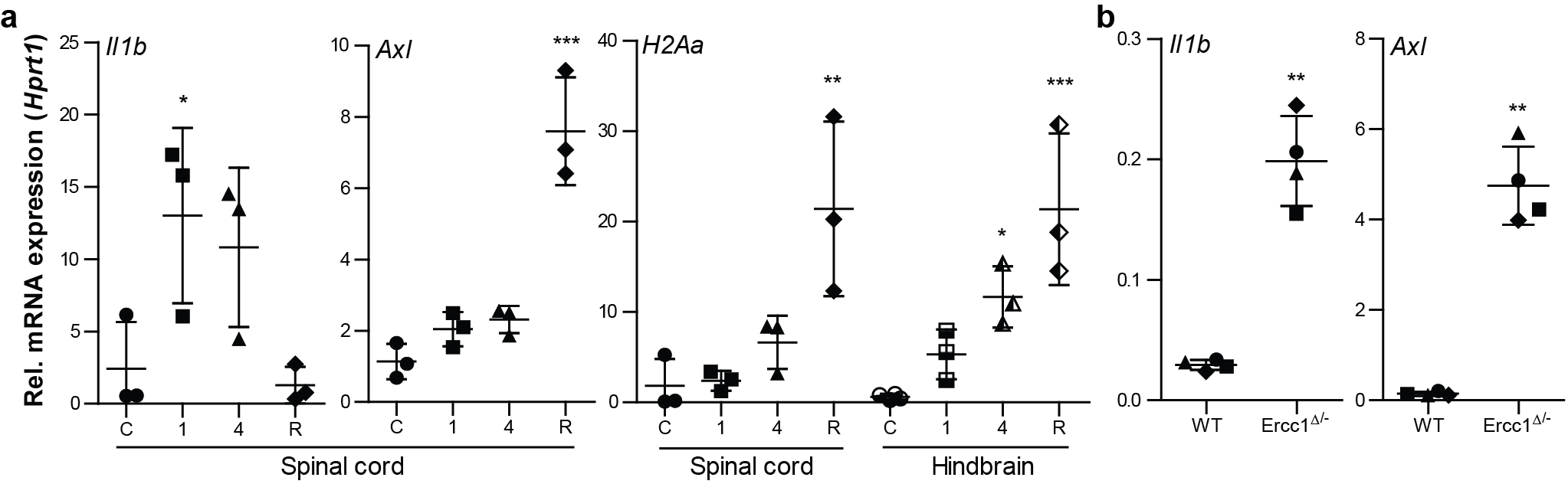


**Supplementary Figure 2.** Acutely isolated adult microglia from Ercc1^∆/-^ and EAE mice exhibit an immune-activated phenotype. **a** Relative gene expression of *Il1b*, *Axl*, and *H2Aa* in acutely isolated adult microglia from spinal cord (*Il1b, Axl, H2Aa*) and hindbrain (*H2Aa*) from EAE control, score 1, score 4 and remission (n=3). **b** Relative gene expression of *Il1b* and *Axl* in acutely isolated adult microglia from whole brain of *Ercc1^∆/-^* mice and WT littermates. Gene expression was measured using RT-qPCR and data are normalised to Hprt1. Statistical analysis conducted was a one-way ANOVA with Dunnett’s test for multiple comparisons (**a**) and a paired Student’s t test (**b**). Error bars indicate mean ± s.d. *p < 0.05, **p < 0.01, ***p < 0.001.


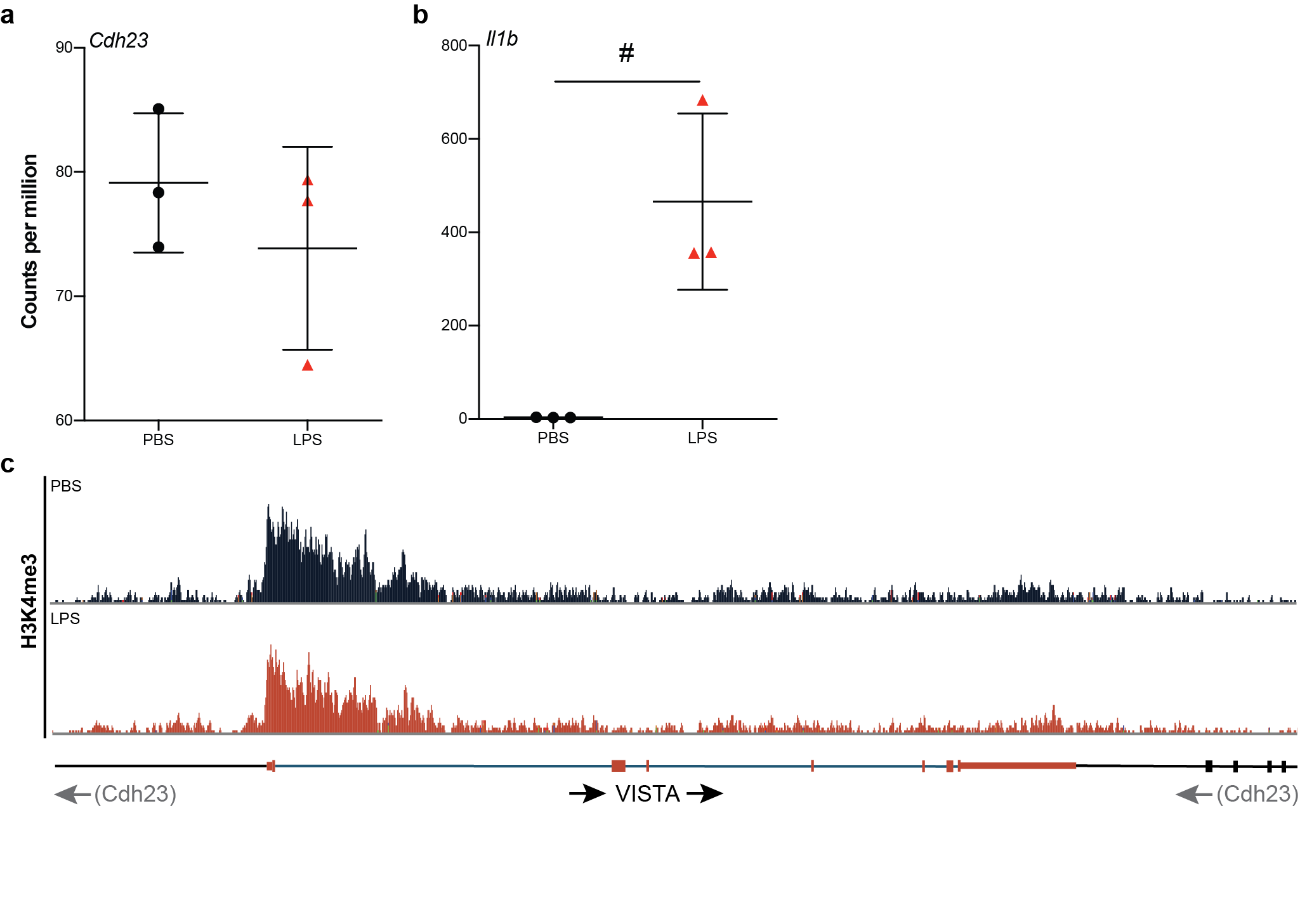
**Supplementary Figure 3.** VISTA H3K4 histone tri-methylation and *Cdh23* and *Il1β* expression in microglia after LPS injection. **a-b** RNA-seq counts per million of *Cdh23* (**a**) and *Il1β* (**b**) mRNA expression (n=3). **c** H3K4me3 histone tri-methylation peaks corresponding to the VISTA gene (n=3). Data is derived from previously generated datasets (X. Zhang et al., n.d.). Error bars indicate mean ± s.d. # = Differential expression (DE) based on RNA-seq analysis.


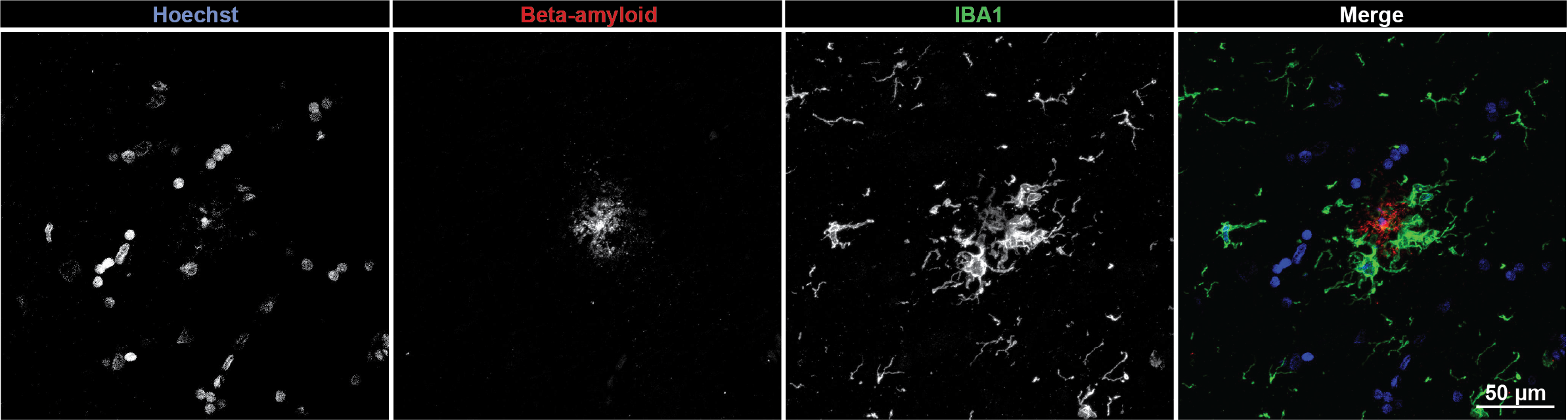


**Supplementary Figure 4.** Microglia cluster around β-amyloid plaque in Alzheimer’s patients. Immunofluorescence staining of β-amyloid (red) and IBA1 (green) in human brain tissue of an Alzheimer’s patient (n=1).
